# Antioxidants Such as Flavonoids and Carotenoids in the Diet of Bogor, Indonesia Residents

**DOI:** 10.3390/antiox10040587

**Published:** 2021-04-11

**Authors:** Nuri Andarwulan, Niken Cahyarani Puspita, Saraswati, Dominika Średnicka-Tober

**Affiliations:** 1 Department of Food Science and Technology, Faculty of Agricultural Technology, IPB University, P.O. Box 220, IPB Darmaga Campus, Bogor 16680, West Java, Indonesia; andarwulan@apps.ipb.ac.id (N.A.); sesilianiken.cp@gmail.com (N.C.P.); ginasaraswati@gmail.com (S.); 2 Southeast Asian Food and Agricultural Science and Technology (SEAFAST) Center, IPB University, IPB Darmaga Campus, Bogor 16680, West Java, Indonesia; 3 Department of Functional and Organic Food, Institute of Human Nutrition Sciences, Warsaw University of Life Sciences, Nowoursynowska 159c, 02-776 Warsaw, Poland

**Keywords:** dietary antioxidants, dietary intake, food frequency questionnaire, food consumption survey, non-communicable diseases

## Abstract

Due to the strong antioxidant activity of flavonoids and carotenoids, daily consumption of these bioactive compounds has the potential for reducing the risk of many chronic and degenerative diseases caused by or contributed to by oxidative stress. Currently, the available research results related to the flavonoid and carotenoid intake in Asian countries are very limited, especially for Indonesian population. The present study was conducted in Bogor City and Bogor District, West Java, Indonesia. Food consumption data was obtained through the food frequency questionnaire (FFQ) method, involving 200 respondents aged 25–65 years old. Flavonoids and carotenoids contents of the consumed food items were determined by referring to the databases developed by the United States Department of Agriculture (USDA), scientific journals, and calculation based on the recipes recorded in the survey. The total flavonoid intake of Bogor adults was estimated as 149.5 mg/day, consisting of 49.4% isoflavones, 24.0% flavonols, 9.4% flavanones, 7.0% flavan-3-ols, 6.0% flavones, and 4.2% anthocyanidins, and was contributed to mainly by legumes (70.7%), vegetables (10.1%), and fruits (7.3%). At the same time, the estimated total carotenoid intake reached 7.6 mg/day, and was contributed to mainly by vegetables (53.9%), fruits (20.2%), and snacks (14.4%), with β-carotene consumed in the highest proportion (49.9%), followed by lycopene (19.9%), lutein and zeaxanthin (13.5%), α-carotene (6.9%), and β-cryptoxanthin (2.6%). The effects of different respondents’ characteristics, such as area of residence (city vs. district), gender (male vs. female), and age (25–40, 41–55, and 56–65 years old) on the flavonoid and carotenoid intake varied widely, due to the differences in the overall consumption patterns of the respective respondents’ groups.

## 1. Introduction

Oxidative stress, which represents an imbalance between free radical production and antioxidant defense in the human body, has an implication for the development of various chronic and degenerative diseases such as cancers and metabolic, cardiovascular, digestive, neurodegenerative, and respiratory diseases [1,2,3,4]. Epidemiological studies suggest that food consumption patterns can influence oxidative stress and its damaging power, in both a positive and a negative way. Hence, dietary strategies for reducing oxidative stress could be a justifiable approach for the prevention of those aforementioned chronic and degenerative diseases [5].

There is strong scientific evidence that a high intake of fruits, vegetables, legumes, and whole grains is associated with a reduced risk of various chronic and degenerative diseases, such as type 2 diabetes, cardiovascular diseases, and cancer [6,7,8]. Besides the benefits of vitamins, minerals, and fiber from plant-based foods, several bioactive constituents such as flavonoids and carotenoids can also improve human health through their action as antioxidants, alleviating oxidative stress [2,9]. Fruits, vegetables, and several plant-based products are known as major sources of flavonoid and carotenoid compounds [10,11]. In addition, carotenoids are also present in other food groups, such as eggs, dairy products, and some types of fish [11].

Flavonoids are a group of natural compounds with variable phenolic structures. They have captivated considerable research interest due to their well-established biological properties, such as antioxidant, anti-inflammatory, and anti-carcinogenic effects [10]. Thanks to the high reactivity of their hydroxyl group, flavonoids scavenge free radicals, which results in lower radical reactivity and stability. Indirectly, flavonoids can also scavenge free radicals by interacting with specific antioxidant enzymes. This can be accomplished through their ability to induce the key enzymes of protection against electrophilic toxicants and oxidative stress, such as NAD(P)H quinone oxidoreductase and S-transferase glutathione. Flavonoids have also shown the ability to chelate metals (iron and copper), thus eliminating causal factors of free radical formation [12]. Data from observational and intervention studies summarized by Vogiatzoglou et al. [13] have shown that habitual flavonoid consumption is associated with reduced risks of cardiovascular diseases, type 2 diabetes, and smoking-related lung cancer.

Carotenoids are a group of naturally occurring lipophilic pigments synthesized by plants, algae, and photosynthetic bacteria, which are recognized as the sources of the yellow, orange, and red colors of many plants [14]. Carotenoid-related health benefits are mainly attributed to their antioxidant activities. They react via different mechanisms, e.g., electron transfer, hydrogen abstraction/reduction, and the formation of carotenoid–radical adducts, depending on the redox potential of the radical species and the structural arrangement of the carotenoid [15]. Dietary carotenoid intake has been correlated with the reduced incidence of several chronic diseases, including cardiovascular diseases, type 2 diabetes, and upper intestinal tract cancer [11].

Estimating the daily intake of flavonoids and carotenoids becomes the first essential step before estimating their potential protective effects against chronic degenerative diseases caused by or contributed to by oxidative stress. The worldwide mean intake of total flavonoids has been reported to be around 400 mg/day, ranging from 150 mg/day to 600 mg/day [10]. A much higher daily flavonoid intake, estimated to reach 1650 mg/day, was reported in the Iranian population [16]. To date, available data of flavonoid intake by the population of Asian countries are very limited, especially for Southeast Asian countries such as Indonesia [10]. The flavonoid intake of the Indonesian population has been reported by Umar and Utari [17], but the estimation was limited to female respondents from the Minangkabau ethnic group (carotenoid intake: 107.3–117.7 mg/day). Estimation of carotenoid intakes of several populations has been reported by some research groups [18,19,20,21,22]. According to various food frequency surveys summarized by Lako et al. [20], the total carotenoid intakes of some populations (US, some European countries, and China) ranged from 4.7 to 17.2 mg/day. The only available data on the total carotenoid intake of the Indonesian population (0.552 mg/day) was been provided by Sefrina et al. [23], who used the food recall 1 × 24 h method. However, the 24 h recall method does not allow for the identification of irregularly consumed foods in a typical diet, therefore the intake of carotenoid compounds estimated with this method can be underestimated.

Thus, this study was conducted to estimate the intake of flavonoids and carotenoids in the Bogor adult population based on the food consumption survey data. The Bogor area (city and district) is well-known as one of the most highly populated areas in Indonesia, where the inhabitants come from various ethnic groups. The results of this study are expected to provide an overview of the food consumption patterns of Bogor adults, estimate the daily intake of flavonoids and carotenoid compounds, and identify the types of food that make largest contribution to the flavonoids and carotenoids intake. In addition, this study can also be the first step for further research related to the effectiveness of flavonoid and carotenoid intake as human health-promoting agents.

## 2. Materials and Methods

### 2.1. Food Consumption Survey

Estimation of flavonoid and carotenoid intake of the Bogor population in this study was based on the food consumption survey conducted by Martianto et al. [24]. The food consumption survey was conducted with a cross-sectional design in Bogor City and Bogor District, West Java, Indonesia, by using the food frequency method. A 2-day semi-quantitative food recall (2 × 24 h) was also conducted as a reference method. Development of the survey instrument in this study was initiated by the harmonization of the questionnaire. Three sets of questionnaires were developed, i.e., respondent characteristics, food recall 2 × 24 h, and food frequency questionnaire (FFQ) (Tables S1–S3). All sets of questionnaires were administered by trained staff. The questionnaire of food recall 2 × 24 h was used to obtain the information about the food consumed by respondents in the 2 × 24 h before interview. The principle of food frequency method is to record the consumption frequency of certain foods or food ingredients during the preceding month. Recapitulation is then performed to get an overview of food consumption patterns. The main information obtained from the survey included name, gender, respondent’s age, and the type and amount of food consumed. The amount of individual food consumption was ascertained using household scale (spoon, bowl, etc.) or other measures commonly used in everyday life and then converted to units of weight (grams). The preparation and food brand was also recorded. The FFQ was developed following the recommendation of Cade et al. [25]. It was initially tested in a trial phase on 10% of respondents before the stage of true questionnaire administration. The food list used in the FFQ is presented in Table S4. Since flavonoid and carotenoid compounds are contained in diverse range of food types, primarily plant-based foods, recording data of consumption frequency was not limited to the list. To date, a validation study regarding FFQ development for assessing flavonoid and carotenoid intake in Indonesia has not been developed by any researchers. Questionnaires used in this study are presented in the Supplementary Materials (Tables S1–S4).

Respondents were included in the survey based on several criteria, such as: age between 25–65 years old, body mass index ranging from 18 to 27, blood pressure between 90/60–140/90 (systole/diastole), health status (healthy/not undergoing treatment of any diseases at the time of the research), and willingness to participate in the study. This study was conducted by referring to the guidelines of the Declaration of Helsinki and was approved by the Institutional Review Board of the School of Medicine, Diponegoro University, Indonesia (protocol code No. 247lEC/FK/RSDK/2012, date of approval July 18, 2012). The minimum required number of samples/respondents was determined using a small sample technique [26], and the formula used was as follows:

(1) $$n=\frac{X^{2}\mathrm{NP}(1-P)}{d^{2}{(N-1)+X}^{2}P(1-P)}$$

Note:

*n* = sample size required

X^2^ = the value obtained from the χ^2^ table for degrees of freedom 1 at the desired confidence level

*N* = population size

P = population proportion (assumed to be 0.50 in order to produce the maximum sample size)

d = margin of error (expressed as a proportion)

The expected level of confidence in the results was 95% with a margin of error of 10%. Data of population size of Bogor City and Bogor District were obtained from the 2010 database of the Central Bureau of Statistics of the Republic of Indonesia. The population of Bogor City in 2010 was 511,600 people, while the population of Bogor District in 2010 was 2,348,609 people. In this study, there were three population categories to be discussed, namely the area of residence (Bogor City and Bogor District), gender (male and female), and age (25–40, 41–55, and 56–65 years old). The minimum number of samples for each category was calculated based on the proportional stratified sampling method, and the calculation was performed according to the following formula:

(2) $$n_{i}=\frac{N_{i}}{N}\times n$$

Note:

*n_i_* = sample size of category *i*

*N_i_* = population size of category *i*

*N* = total population size

*n* = total sample size

In brief, this survey involved 200 respondents aged 25–65 years old, covering 100 Bogor City residents (50 males and 50 females) and 100 Bogor District residents (50 males and 50 females). The detailed information regarding sample size for each category is provided in Table 1.

### 2.2. Estimation of Flavonoids and Carotenoids Intake

Information on the flavonoids and carotenoids level contained in each food item consumed by the Bogor population was obtained from secondary databases, such as the USDA database and several scientific papers. The USDA Database for the Flavonoid Contents of Selected Foods, Release 3.1 in 2013 [27] and the USDA Database for the Isoflavone Contents of Selected Foods, Release 2.0 in 2008 [28] were used as main databases for determining food flavonoids content, while the USDA National Nutrient Database for Standard Reference, Release 27 in 2014 [29] was used as main database of food carotenoids content.

Flavonoid levels in this study are expressed as total flavonoid content and the content of particular subclasses of flavonoids. The subclasses of flavonoids included anthocyanidins, flavan-3-ols, flavanones, flavones, flavonols, and isoflavones. Although the flavonoid content of food materials or products could be obtained from various sources including USDA food databases and other scientific publications, there was a possibility of secondary data being unavailable for some food items consumed by respondents. For those cases, the estimation techniques for calculating unavailable values of flavonoid content was performed by referring to Bhagwat et al. [30].

Carotenoid content of foodstuffs discussed in this study included six carotenoid compounds generally found in high concentrations in human blood samples, namely α-carotene, β-carotene, β-cryptoxanthin, lutein and zeaxanthin, and lycopene [11]. The contents of lutein and zeaxanthin are usually expressed as a mixture because of the difficulty in separating these compounds. There are no specific provisions for determining the food carotenoid content based on the existing databases, but as applied in the flavonoid content determination, in the case of foodstuffs for which secondary data on carotenoid content are unavailable, the carotenoid content can be estimated based on available data for foods having similar characteristics.

The flavonoid and carotenoid content of multi-ingredient foods were calculated as a sum of the flavonoid or carotenoid content of the constituent ingredients, by considering the proportional factor of each ingredient to the total weight of food. The ingredients’ proportion in certain food items was calculated based on food recipe information, which was previously recapitulated through the food frequency survey. The equation used for the calculation of the flavonoid and carotenoid contents in the multi-ingredients foods is as follows:

(3) $${\mathrm{XC}}_{i}={\mathrm{XC}}_{A}{\times p}_{Ai}{+\mathrm{XC}}_{B}{\times p}_{Bi}+\ldots$$

Note:

XC_*i*_: flavonoid/carotenoid content of food *i*

XC_A_: flavonoid/carotenoid content of ingredient A based on the database

p_A*i*_: proportion of ingredient A in food *i*, expressed as a percentage or ratio (weight of ingredient A/total weight of food *i*)

XC_B_: flavonoid/carotenoid content of ingredient B based on the database

p_B*i*_: proportion of ingredient B in food *i*, expressed as a percentage or ratio (weight of ingredient B/total weight of food *i*)

The flavonoid and carotenoid intake from certain food items was calculated based on the following equation:

(4) $${\mathrm{XI}}_{i}=w_{i}\times \frac{{\mathrm{XC}}_{i}}{100}\times \frac{p_{i}}{100}$$

Note:

XI_*i*_: flavonoid/carotenoid intake from food *i*

w_*i*_: weight of consumed food *i* (gram)

XC_*i*_: flavonoid/carotenoid content of food *i* based on the database (mg/100 g for flavonoid, µg/100 g for carotenoid)

p_*i*_: edible portion

The average daily intake of flavonoids and carotenoids from certain food items was calculated based on the following equation:

(5) $${\mathrm{DXI}}_{i}=\frac{{\mathrm{XI}}_{i}{\times f}_{i}}{30}$$

Note:

DXI_*i*_: daily intake of flavonoid/carotenoid from food *i*

XI_*i*_: flavonoid/carotenoid intake from food *i*

f_*i*_: consumption frequency of food *i* in a month (1 month is assumed as 30 days)

The daily intake of flavonoids/carotenoids for each individual was calculated by summing up the entire flavonoids/carotenoids intake from all daily consumed foods. Data of flavonoids/carotenoids intake were re-categorized into 11 food groups, i.e., (1) beverages; (2) fruits and fruit products; (3) herbs, spices, and condiments; (4) cereals and cereal products; (5) eggs and egg products; (6) fish and fish products; (7) legumes, legume products and nuts; (8) meat and meat products; (9) snack foods; (10) supplements; and (11) vegetables and vegetable products.

### 2.3. Statistical Analyses

Data on food consumption, flavonoids, and carotenoids intake are expressed as grams/milligrams/micrograms per person per day. Effects of different respondents’ categories (area of residence, gender and age) on the studied intake parameters were analyzed statistically by one-way analysis of variance (ANOVA) followed by a *t*-test. *p* < 0.05 was considered statistically significant. Statistical analyses were performed in SPSS software version 20 (SPSS Inc., Chicago, IL, USA).

## 3. Results and Discussion

### 3.1. Daily Consumption Pattern of Bogor Adult Residents

In this study, various foods (270 food items) consumed by Bogor residents were categorized into 11 food groups based on their main ingredients (Table S5). Although the dishes derived from meat, fish and seafood were not expected to contain flavonoids, those types of food groups may also contribute to the flavonoid intake when considering the presence of other, plant-based ingredients in their composition. The consumption pattern and the contribution of each of the 11 food groups to the total food consumption of Bogor residents is presented in Table 2. The average food consumption of the residents of Bogor City and District was estimated as 892 g/person/day. The food groups with the largest contribution to the total food consumption were cereals and cereal products (44.6%), followed by vegetables and vegetable products (15.8%) and fruits and fruit products (11.9%). Unlike other food groups, cereals and cereal products were consumed by all respondents (100%). This is related to the common consumption pattern of Indonesian people, who rely on rice (cereals) as the staple food. In addition, this study showed that vegetables and their processed products, fruits and fruit products, legumes and legume products, and snacks were consumed by a high percentage of the studied population, while food supplements were only consumed by 5% of respondents.

The average consumption of fruit and of vegetables and their processed products by the studied population was estimated as 247.1 g/person/day, which is in line with the results reported by Setyowati et al. (2018) for Jakarta residents [31]. The fruit and vegetables that respondents usually consumed were bananas (local banana), cabbage, spinach, and water cress. The recommended fruit and vegetables intake, excluding potatoes and other starchy tubers, according to the current recommendations of the World Health Organization (WHO) and the Food and Agriculture Organization of the United Nations (FAO) is a minimum of 400 g (or five 80 g portions) a day [32].

Such amount of fruit and vegetables in a habitual diet is considered as an important contributor to the prevention of chronic diseases, such as cancers, cardiovascular diseases, diabetes, obesity, as well as for the prevention and alleviation of several micronutrient deficiencies, especially in less developed countries [32]. The low intake of products from these important food categories by the Bogor population is caused by their limited economic accessibility. High prices of fruits and vegetables (especially fruits) result from (a) inefficient agricultural and horticultural practices, (b) long distances between the location of the main production areas and the target market (high transportation costs), and (c) lack of efficient logistics causing high food losses. It should be pointed that low intake of vegetables and fruit is listed as one of the top ten risk factors for mortality globally. According to the Global Burden of Disease Study [33], 1.8 million deaths/year worldwide can be attributed to low consumption of vegetables and 3.4 million to diets low in vegetables. Although vegetables and fruit consumption has been extensively promoted worldwide in the recent decades, per capita consumption is still estimated to be 20 to 50% below the minimum daily recommended level of 400 g [34]. Relevant actions taken to increase fruit and vegetables consumption by the Indonesian population could be therefore considered as important initiatives for improving public health.

The results on the average consumption of each food group by the respondents depending on their area of residence, gender, and age are shown in Table 3. None of the three factors (area of residence, gender, and age) were shown to significantly affect the total food consumption. The *t*-test results on each food group’s consumption also showed that there were no significant differences between Bogor residents representing the two different areas (city vs. district). Gender-related differences were identified in the case of a number of food groups, i.e., female respondents consumed significantly higher amounts of herbs, spices, and condiments, as well as supplements, compared to the male respondents, while male respondents consumed significantly more cereals and cereal products. There was also a trend (*p* = 0.083) towards higher consumption of vegetables and vegetable products in the female group. Significant age-related differences were noticed only in the case of cereals and cereal products, which were consumed in higher amounts by two younger (25–40 y and 41–55 y) groups compared to the oldest (56–65 y) respondent group.

Some significant differences were also identified when a more in-depth analysis was carried out for each respondent’s subcategory (Table S6), i.e., ANOVA results on female respondents representing different age groups showed that women aged 56–65 years old tended to consume significantly lower amounts of food (696.56 g/person/day) than women in the two other age groups (25–40 and 41–55 years old). In Bogor City, the total food consumption of respondents aged 41–55 years old was significantly higher than that of respondents in the 25–40 years old and 56–65 years old groups. This can be due to the tendency of increasing food consumption at the peak of the productive age (age 35–44 years), and a significant decrease when a person has passed the productive age, especially starting from the age of 50 years and over [35].

Specific observation on the male respondents representing different age groups showed that men aged 41–55 years old consumed significantly higher amounts of beverages than men in other age groups. Moreover, consumption of cereals and cereal products by male respondents was decreasing with age (lowest in the 56–65 years old group), while consumption of vegetables and vegetable products was increasing with age (highest in the 56–65 years old group). Interestingly, in the case of female respondents, the consumption of cereals and cereal products, but also vegetables and vegetable products and snacks was decreasing with age (lowest in the 56–65 years old group). At the same time the consumption of eggs and their processed foods by female respondents aged 41–55 years old was significantly higher than in other age groups of women (but this difference was significant only in the case of district area).

It is important to point that, in case of female respondents, the consumption of products from groups such as vegetables, fruits, legumes, and supplements was decreasing with age (was lowest in the oldest age group—56–65 y), while risk of many diseases is known to be growing, and the bioavailability of many nutrients is considerably decreasing with age [36]. Thus, people from older age groups should be considered as an important target for actions towards the increase of fruit and vegetables consumption and/or dietary supplementation.

The gender-related differences in the consumption level of different food groups were more evident in the city area compared to the district area of Bogor: females in the city area consumed significantly more beverages; more herbs, spices and condiments; and more supplements, but less cereals and cereal products than males from the same area. Similar, although not as significant trends, were observed in the district area, except for beverages, for which the results recorded for the district and city area were opposite (in the district area the consumption of beverages was significantly higher in the group of males compared to female respondents).

Consumption of meat and meat products by females in the city area was decreasing with age (lowest in the 56–65 years old group). Although there was a similar tendency in the male group in the same area, the difference was not significant. Male respondents aged 56–65 years old in the city area consumed fish and fish products in highest amount compared to men from other age groups.

In the district area, consumption of eggs and egg products was higher in the age group of 41–55 years old (as already described above), but this difference was in fact statistically significant only in the case of female respondents. The men aged 56–65 years old from the district area consumed the highest amounts of legumes and of vegetables and their processed products compared to men from other age groups in the same area, but a similar trend was not observed in the case of women from the same area (Table S6).

### 3.2. Estimated Daily Flavonoid Intake of Bogor Adult Residents

Table 4 shows the data sources used for determination of flavonoid and carotenoid contents in 270 food items consumed by Bogor residents. Total flavonoid and carotenoid content of each food category and examples of calculations of their content from multi-ingredient foods are shown in Tables S7–S10. Some of the food items were assigned as not containing flavonoids (3 food items) or carotenoids (9 food items). The levels of flavonoids and carotenoids contained in several foods could not be determined (27 unknown values for flavonoids and 17 unknown values for carotenoids) due to limited information in the secondary databases. A list of food items with unknown flavonoid and carotenoid content is available in Tables S11 and S12.

These study results showed that the total consumption of flavonoids by Bogor residents (both city and district) was 149.5 mg/day (Table 5). As much as 70.7% of the total flavonoids consumed by all respondents were sourced from legumes and legume products. Other food groups significantly contributing to the total flavonoid intake included vegetables and vegetable products (10.1%) and fruits and fruit products (7.3%). At the same time, food groups 6 (fish and fish products) and 10 (supplements) only provided a negligible contribution to the total daily flavonoids intake. Our findings are in accordance with the statement of Bhagwat et al. [30], affirming that the main sources of flavonoid compounds, besides isoflavones, are vegetables and fruits, while the main sources of isoflavones are legumes, especially soy-based food products.

As already described, according to the data on the consumption patterns of Bogor adults, the foods consumed in the highest amount were cereals and their processed products. However, this group contained relatively low amounts of flavonoids, and thus the contribution of this food group to the total daily intake of flavonoids only reached 2.4%. On the other hand, consumption of legumes and their products only accounted for 9.1% of the total food consumption, but products from this food group were considered to be the main contributors to the flavonoid intake because of their high content of flavonoid compounds. Isoflavones were identified as the most consumed flavonoid compounds (49.4% of the total daily flavonoid intake), followed by flavonols (24.0%), flavanones (9.4%), flavan-3-ols (7.0%), flavones (6.0%), and anthocyanidins (4.2%). The types of food that greatly contributed to the isoflavone intake were tempeh, tofu, and other soy-based products such as, e.g., tauco.

Table 6 shows the daily intake of flavonoid subclasses for different respondents’ categories (based on area of residence, age, gender). The area of residence (city vs. district) was observed to significantly affect the anthocyanidin and isoflavone intake. The isoflavone intake of Bogor City residents was higher than that of Bogor District residents. It was related to the higher intake of legumes and legume products by Bogor City residents.

Fruits and fruit products were considered to be the main contributor to anthocyanidin intake. Although the fruit consumption of Bogor City and District residents did not differ significantly, the significant difference in the anthocyanidin intake could be associated with the different types of fruit consumed by both populations. Grapes, strawberries, blueberries, and dragon fruit are reported to contain high levels of anthocyanidins [30], so these fruits might have given high contribution to the anthocyanidin intake. Detailed comparison of flavonoid intake between several respondent subcategories are provided in Table S13.

The daily flavonoid intake may vary by geographical region due to different factors, such as dietary patterns, the flavonoid classes included, the food consumption survey methods, the food composition database used, and the method of expression. Escovar-Cevoli et al. [10] summarized data on the mean intake of flavonoids worldwide, which ranged between 150–600 mg/day, expressed as aglycones. They also added that the main food items contributing to the daily flavonoid intake included black tea, fruits (i.e., berries, apples), red wine, cocoa, beans, soy, tofu, and vegetables (i.e., onions). Chun et al. [37] reported the estimated average daily total flavonoid intake of U.S. adults to be 189.7 mg/day, which was mainly from flavan-3-ols (83.5%), followed by flavanones, flavonols, anthocyanidins, flavones, and isoflavones. Food items mainly contributing to the flavonoid intake of U.S. adults included tea, citrus fruit juices, wine, and citrus fruits. These findings were not in accordance with the present study because of the marked differences in the consumption patterns between the two different observed populations. A higher average intake of total flavonoids (428.0 mg/day) was found in the population of European adults, as reported by Vogiatzoglou et al. [13], with flavan-3-ols as the main contributors. The total flavonoid intakes of the Chinese and South Korean populations were about 225–320 mg/day [10]. While Sohrab et al. [16] found that Iranian adults consumed flavonoid compounds in a much higher amount, reaching 1650 mg/day. This high intake is due to the elevated consumption of black tea in those Middle Eastern populations. Currently, the recommended daily intake of total flavonoids or subclasses of flavonoids is still not well defined, therefore, statements regarding the adequacy of daily flavonoid intake of the Bogor adult population cannot be formed yet.

### 3.3. Estimated Daily Carotenoid Intake of Bogor Adult Residents

The estimated daily carotenoid intake of Bogor residents in this study was 7578 µg/day or 7.6 mg/day. The contribution of each food group to the total carotenoid intake can be seen in Table 7. Vegetables and vegetable products were observed as the main contributors (53.9%) to the total carotenoid intake, followed by fruits and fruit products (20.2%) and snacks (14.4%). Many vegetables have been recognized as rich sources of carotenoid compounds. Fruits and their processed products, primarily those possessing colors in the yellow to red spectrum, are also known as a good source of carotenoids [38]. A considerably high contribution of snacks to the estimated total carotenoid intake is related to the fact that many of the snacks consumed regularly by Indonesians are based on/contain fruit and vegetables.

β-carotene was found as the main carotenoid compound consumed by all respondents. As much as 49.9% of total carotenoid intake was from β-carotene, followed by lycopene (19.9%), lutein and zeaxanthin (13.5%), α-carotene (6.9%), and β-cryptoxanthin (2.6%). The main food sources of β-carotene in this study were vegetables and their processed products.

Table 8 shows that total carotenoid intake (and especially β-carotene intake) of Bogor District residents was significantly higher than that of Bogor City residents (*p* < 0.05). This can be due to the higher consumption of vegetable-based foods by Bogor District residents. At the same time, the gender and age of the respondents seemed not to significantly influence the total carotenoid intake of the Bogor population. Detailed comparison of carotenoid intakes between several respondent subcategories is provided in Table S14.

Total carotenoid intake found in this study was lower than reported in some previous reports on the carotenoid intakes of several populations, for example: 14 mg/day by European adults from the UK, Ireland, Spain, France and the Netherlands [18], 10.69–22.61 mg/day by Irish adults [19], and 20 mg/day by the Fijian population [20]. However, the total carotenoid intake of Bogor adults was higher than that of overweight and obese Dominican subjects, which only reached 6.36 mg/day [22] and the female population in the U.S., who only consumed 6.0 mg of total carotenoids/day [21]. In line with the present study, several aforementioned studies also reported the role of β-carotene as the main contributor to the daily total carotenoid intake. Carrot was considered to be the main source of β-carotene intake in the European population [18].

Carotenoids are known as one of the pigment groups found in plants. Three of the carotenoid compounds investigated, namely α-carotene, β-carotene and β-cryptoxanthin, can be converted into retinol, and thus are referred to as provitamin A carotenoids. At the same time lutein, zeaxanthin, and lycopene are considered to be non-provitamin A carotenoids [14]. The daily intake of provitamin A carotenoids may contribute to the daily vitamin A intake. The recommended dietary allowance (RDA) or adequate intake (AI) for carotenoids is not established yet, but the US Food and Drug Administration (FDA) has set the RDA for vitamin A to be 900 µg retinol activity equivalents (RAE) per day [39]. Detailed information regarding retinol activity equivalent ratios for preformed vitamin A and provitamin A carotenoids have been provided by Hidgon [14]. Although provitamin A carotenoids intake can protect consumers from vitamin A deficiency, the deficiency symptoms can be also avoided in people consuming low-carotenoid diets in the case of an adequate vitamin A intake [40].

As previously described, habitual flavonoid and carotenoid intakes are associated with the reduced risks of several chronic diseases, primarily cardiovascular disease (CVD) and type 2 diabetes mellitus [11,13]. Currently, chronic diseases, also termed as non-communicable diseases (NCDs), are reported to be the leading causes (about 71%) of death in the world. Over 85% of those premature deaths occur in low- and middle-income countries [41]. Indonesia is also experiencing a dramatic escalation in the problem of NCDs. This is evidenced by an increase in the prevalence of NCDs from year to year based on the results of the National Basic Health Research in 2007, 2013, and 2018 [42]. For example, the results of the National Basic Health Research in 2007 and 2013 showed that the prevalence of stroke increased from 8.3‰ in 2007 to 12.1‰ in 2013. Increased prevalence is also observed in type 2 diabetes mellitus and hypertension, i.e., from 1.1% and 7.6% in 2007, respectively, to 2.1% (diabetes mellitus) and 9.5% (hypertension, only based on medical doctors’ diagnoses) in 2013 [43]. Coronary heart disease (CHD), stroke, and diabetes mellitus are identified as the main NCDs in Indonesia. In addition, hypertension and obesity are recognized as the main risk factors for the incidence of those three main NCDs. According to the data of the National Basic Health Research in 2013 [43], the prevalence of diabetes mellitus, hypertension, and CHD were found to be higher in women than men. At the same time, the prevalence of stroke in men and women were similar. The prevalence of CHD, stroke, heart failure, diabetes mellitus, and hypertension tended to increase with age. Furthermore, urban communities tended to have a higher prevalence of diabetes mellitus, CHD, stroke, and hypertension than rural communities. Health profile data of West Java Province, Indonesia in 2012 [44] showed that the prevalence of diabetes mellitus in Bogor City is higher than in Bogor Regency, while the prevalence of hypertension was found to be higher in Bogor Regency than in Bogor City.

As previously mentioned, some significant differences in the carotenoid intake can be found between respondents from two different areas of residence. Respondents from Bogor Regency (rural area) consumed more carotenoids than Bogor City respondents (urban). In the case of the flavonoid subclasses’ intake, respondents from Bogor city consumed higher amounts of anthocyanidins, flavones, and isoflavones. Furthermore, although not significant, the total flavonoid and carotenoid intake in female respondents tended to be higher than that of male respondents. Even though the results of the National Basic Health Research [43] show that the prevalence of various NCDs is closely related to the area of residence (rural vs. urban), age (young vs. elderly), and gender (male vs. female), the association between dietary patterns (including total flavonoid and carotenoid intake) and the reported facts regarding the NCDs prevalence in Indonesia has not been clearly seen yet. This can be explained by the fact that the dietary patterns, including the intake of flavonoids and carotenoids, are among a number of factors influencing the development of NCDs [45], such as, e.g., age, gender, physical (in)activity, and tobacco and alcohol use.

### 3.4. Limitations of the Study

The presented study had several limitations. The estimation of phytochemical intakes was based on the secondary data of carotenoids and flavonoids content in foods. Chemical analyses of the antioxidants’ concentrations and profiles in particular food products within the study would undoubtedly provide much more precise information, and thus would allow for a more exact estimation of the intakes of particular carotenoid and flavonoid compounds. This would also allow for a much broader and relevant discussion on the potential health-promoting effectiveness of particular food groups/food items (considering different antioxidant potential and other activities of particular flavonoid and carotenoid compounds). It should also be pointed that the consumed foods (i.e., vegetables and fruits) represent a wide range of varieties (genotypes), are harvested in different climate zones/seasons, are grown in various agronomic conditions, and undergo various postharvest handling methods, and therefore may significantly vary with regard to the content of specific plant secondary metabolites [46,47,48]. Another important factor known to have a significant impact on the phenolic and carotenoid profiles of foods, and not taken into consideration in the present study, is food processing, including methods and specific conditions (e.g., temperature and time of thermal processing, etc.) [49,50,51,52]. Finally, the study would undoubtedly benefit from the measurement of antioxidant levels in the plasma of respondents, and from correlation analysis between plasma antioxidants and the presented study outcomes (intakes of the targeted groups of phytochemicals).

## 4. Conclusions

From this study, we found that the total flavonoid and carotenoid intakes of Bogor adults reached 149.5 mg/day and 7.6 mg/day, respectively. Legumes and their processed products were considered to be the main contributor to the total flavonoid intake. At the same time, vegetables and vegetable products were identified as the main source of dietary carotenoid compounds. Among all considered subgroups of flavonoids, isoflavones were consumed by Bogor residents in the highest proportion, while as much as 49.9% of the total carotenoid intake was contributed by β-carotene.

Some significant differences in the carotenoid intake were found between respondents from two different areas of residence. Respondents from the rural area consumed more carotenoids than Bogor city residents. In the case of the flavonoid subclasses’ intake, respondents from Bogor city consumed higher amounts of anthocyanidins, flavones, and isoflavones. Furthermore, although not significant, the total flavonoid and carotenoid intake of female respondents tended to be higher than that of male respondents.

The study provides an important overview of the food consumption patterns of Bogor adults, as well as of the daily intake of important antioxidants such as flavonoids and carotenoids, and identifies the types of food that make the largest contributions to the flavonoid and carotenoid intake. It also points out the generally low fruit and vegetable intake by the studied population, and thus confirms a strong need for relevant actions to be taken towards the increase in the consumption level of these important food groups, rich in health-promoting phytochemicals with strong antioxidant properties. The results of this study can be used as baseline information for several stakeholders, especially for determining public health-related policies. Moreover, this study can also serve as the first step for further research towards documenting the health-promoting and protective effects of the intake of antioxidants such as flavonoids and carotenoids.

## Figures and Tables

**Table 1:** The sample size for each category and subcategory of respondents.

Category and Subcategory	Total Population	Proportion (%)	Minimum Required Sample ^1^	Available Sample	Sample Adequacy
Bogor City	511,600		96	100	Yes
Age 25–40 y	233,694	45.68	44	46	Yes
Age 41–55 y	165,994	32.45	31	33	Yes
Age 56–65 y	111,912	21.87	21	21	Yes
*Male*	248,403	48.55	47	50	Yes
Age 25–40 y	95,924	18.75	18	19	Yes
Age 41–55 y	85,049	16.62	16	18	Yes
Age 56–65 y	67,430	13.18	13	13	Yes
*Female*	263,197	51.45	49	50	Yes
Age 25–40 y	137,770	26.93	26	27	Yes
Age 41–55 y	80,945	15.82	15	15	Yes
Age 56–65 y	44,482	8.69	8	8	Yes
Bogor District	2,348,609		96	100	Yes
Age 25–40 y	1,342,475	57.16	55	56	Yes
Age 41–55 y	685,581	29.19	28	30	Yes
Age 56–65 y	320,553	13.65	13	14	Yes
*Male*	1,211,731	51.59	50	50	Yes
Age 25–40 y	722,438	30.76	30	30	Yes
Age 41–55 y	342,505	14.58	14	14	Yes
Age 56–65 y	146,788	6.25	6	6	Yes
*Female*	1,136,878	48.41	46	50	Yes
Age 25–40 y	620,037	26.40	25	26	Yes
Age 41–55 y	343,076	14.61	14	16	Yes
Age 56–65 y	173,765	7.40	7	8	Yes
**Total Bogor Population**	2,860,209		96	200	Yes
Age 25–40 y	1,576,169	55.11	53	102	Yes
Age 41–55 y	851,575	29.77	29	63	Yes
Age 56–65 y	432,465	15.12	15	35	Yes
*Male*	1,460,134	51.05	49	100	Yes
Age 25–40 y	818,362	28.61	27	45	Yes
Age 41–55 y	427,554	14.95	14	34	Yes
Age 56–65 y	214,218	7.49	7	21	Yes
*Female*	1,400,075	48.95	47	100	Yes
Age 25–40 y	757,807	26.49	25	57	Yes
Age 41–55 y	424,021	14.82	14	29	Yes
Age 56–65 y	218,247	7.63	7	14	Yes

^1^ Minimum required sample size was calculated based on the equation described by Krejcie and Morgan (1970) [26].

**Table 2:** Average consumption of each food group by Bogor adult residents.

No.	Food Group	Daily Consumption Per Person (g/Person/Day)					Number of Eaters (*n* = 200)	% Eaters
		Mean	Min	Max	95% Tile	% Consumption		
1	Beverages ^1^	16.20	0.00	251.07	70.88	1.8	131	65.5
2	Fruits and fruit products	106.13	0.00	601.33	310.23	11.9	197	98.5
3	Herbs, spices, and condiments	13.01	0.00	119.00	50.10	1.5	170	85.0
4	Cereals and cereal products	397.94	21.87	1097.07	640.44	44.6	200	100.0
5	Eggs and egg products	9.72	0.00	88.00	49.20	1.1	74	37.0
6	Fish and fish products	1.86	0.00	35.20	10.80	0.2	48	24.0
7	Legumes and legume products	81.55	0.00	386.07	201.75	9.1	197	98.5
8	Meat and meat products	28.40	0.00	240.95	93.60	3.2	159	79.5
9	Snacks	96.15	0.00	536.47	251.67	10.8	197	98.5
10	Supplements	0.08	0.00	5.00	0.03	0.0	10	5.0
11	Vegetables and vegetable products	140.93	0.00	631.15	347.41	15.8	199	99.5
	Total	891.99						

^1^ excluding drinking water.

**Table 3:** Average consumption of each food group by the respondents depending on their area of residence, gender, and age.

No	Food Group	Daily Consumption Per Person (g/Person/Day)									
		Area of Residence		*p*-Value ^1^	Gender		*p*-Value	Age Group (Years Old)			*p*-Value
		City	District		Male	Female		25–40	41–55	56–65	
1	Beverages ^2^	18.60	13.81	0.239	14.20	18.21	0.299	14.00	19.70	16.32	0.455
2	Fruits and fruit products	111.91	100.35	0.459	103.59	108.67	0.745	104.34	109.33	105.59	0.958
3	Herbs, spices, and condiments	13.94	12.08	0.413	8.88	17.13	**0.004**	14.99	11.06	10.74	0.389
4	Cereals and cereal products	383.21	412.68	0.183	424.32	371.56	**0.011**	421.29	394.89	335.41	**0.017**
5	Eggs and egg products	10.86	8.58	0.404	9.57	9.87	0.912	8.27	13.91	6.43	0.067
6	Fish and fish products	2.35	1.37	0.159	1.74	1.98	0.723	1.65	1.43	3.26	0.144
7	Legumes and legume products	84.54	78.56	0.549	77.39	85.71	0.374	77.85	87.01	82.50	0.683
8	Meat and meat products	32.68	24.13	0.068	28.89	27.92	0.841	31.17	28.12	20.86	0.294
9	Snacks	98.38	93.92	0.715	99.20	93.11	0.608	100.69	90.52	93.07	0.722
10	Supplements	0.12	0.05	0.323	0.00	0.16	**0.019**	0.10	0.11	0.00	0.507
11	Vegetables and vegetable products	132.46	149.41	0.265	129.11	152.76	0.083	136.64	151.41	134.59	0.605
	Total	889.04	894.94	0.912	896.89	887.09	0.849	910.98	907.48	808.77	0.299

^1^*p*-Value < 0.05 means that the consumption level of a particular food category differs significantly between the respective population groups; ^2^ excluding drinking water. Bold numbers: *p*-Value < 0.05.

**Table 4:** Data sources for the determination of flavonoid and carotenoid contents of various foods (270 food items in total) consumed by respondents.

Data Sources for Determining Flavonoids and Carotenoids Content	Number of Food Items			
	Flavonoids	(%)	Carotenoids	(%)
Secondary data, such as the USDA database and several scientific journals	101	37.4	109	40.4
Assigned as not containing flavonoids or carotenoids	3	1.1	9	3.3
Calculated based on recipes	139	51.5	135	50.0
Not available	27	10.0	17	6.3
Total	270	100	270	100

**Table 5:** Estimated daily flavonoid intake of Bogor adult residents.

No	Food Group	Flavonoid Intake (mg/person/day)						
		Antho-Cyanidins	Flavan-3-ols	Flavanones	Flavones	Flavo-nols	Isoflavones	Total
1	Beverages ^1^	0.000	1.614	0.000	0.000	0.097	0.001	1.712
2	Fruits and fruit products	1.607	1.940	5.909	2.730	0.478	0.000	10.976
3	Herbs, spices, and condiments	0.088	0.013	0.000	0.423	0.474	0.021	1.364
4	Cereals and cereal products	0.056	0.016	0.082	0.096	0.763	0.425	3.622
5	Eggs and egg products	0.002	0.000	0.000	0.013	0.008	0.005	0.027
6	Fish and fish products	0.000	0.000	0.000	0.000	0.003	0.000	0.003
7	Legumes and legume products	0.109	0.159	0.000	0.117	0.441	22.380	105.769
8	Meat and meat products	0.042	0.011	0.036	0.039	0.348	0.001	0.447
9	Snacks	0.264	0.422	0.000	0.021	0.181	9.175	10.506
10	Supplements	0.000	0.000	0.000	0.000	0.001	0.000	0.001
11	Vegetables and vegetable products	0.542	0.401	0.115	0.457	12.854	0.192	15.093
	Total	2.709	4.575	6.142	3.896	15.647	32.200	149.520

^1^ excluding drinking water.

**Table 6:** Daily intake of flavonoid subclasses for different respondents’ categories (based on area of residence, age, gender).

No	Flavonoid Subclass	Flavonoid Intake (mg/person/day)									
		Area of Residence		*p*-Value ^1^	Gender		*p*-Value	Age group			*p*-Value
		City	District		Male	Female		25–40 y	41–55 y	56–65 y	
1	Anthocyanidins	3.072	2.347	**0.026**	2.663	2.756	0.795	2.549	2.761	3.083	0.584
2	Flavan-3-ols	5.173	3.978	0.118	4.392	4.758	0.577	3.824	5.434	5.217	0.100
3	Flavanones	6.73	5.555	0.428	6.491	5.793	0.606	6.750	6.110	4.428	0.500
4	Flavones	2.237	1.861	0.079	4.374	3.418	0.393	4.785	2.801	3.275	0.310
5	Flavonols	16.095	15.199	0.644	14.364	16.929	0.166	14.477	18.045	14.74	0.198
6	Isoflavones	28.847	26.553	**0.047**	33.881	30.519	0.391	30.914	33.135	34.262	0.765
	Total	195.48	124.775	0.113	143.558	155.481	0.662	161.246	140.995	130.692	0.695

^1^*p*-Value < 0.05 means that the daily intake of a particular flavonoid subclass differs significantly between respective population groups. Bold numbers: *p*-Value < 0.05.

**Table 7:** Estimated daily carotenoid intake of Bogor residents.

No	Food Group	Carotenoid Intake (µg/person/day)					
		α-Carotene	β-Carotene	β-Cryptoxanthin	Lutein and Zeaxanthin	Lycopene	Total
1	Beverages ^1^	1.850	6.798	0.035	2.139	7.492	18.314
2	Fruits and fruit products	12.702	235.890	182.477	67.594	1030.459	1529.122
3	Herbs, spices, and condiments	10.005	30.833	1.008	16.937	102.968	160.985
4	Cereals and cereal products	17.951	89.377	9.847	64.089	165.657	346.916
5	Eggs and egg products	0.225	2.878	0.857	41.855	1.109	46.923
6	Fish and fish products	0.000	0.000	0.000	0.000	0.000	0.032
7	Legumes and legume products	5.515	212.954	0.717	35.178	23.343	277.707
8	Meat and meat products	0.484	6.054	0.373	4.121	12.708	23.740
9	Snacks	205.965	737.708	3.532	39.045	101.765	1088.016
10	Supplements	0.001	0.185	0.002	0.001	0.383	0.572
11	Vegetables and vegetable products	309.128	2751.503	11.129	832.651	181.662	4086.076
	Total	563.826	4074.181	209.976	1103.610	1627.547	7578.404

^1^ excluding drinking water.

**Table 8:** Daily intake of individual carotenoid compounds by different respondents’ categories (based on area of residence, age, gender).

No	Carotenoid	Carotenoid Intake (mg/person/day)									
		Area of Residence		*p*-Value ^1^	Gender		*p*-Value	Age Group			*p*-Value
		City	District		Male	Female		25–40 y	41–55 y	56–65 y	
1	α-carotene	0.518	0.610	0.289	0.537	0.590	0.515	0.584	0.592	0.454	0.466
2	β-carotene	3.190	4.959	**0.000**	3.814	4.334	0.276	3.979	4.307	3.932	0.795
3	β-cryptoxanthin	0.201	0.218	0.659	0.176	0.244	0.081	0.216	0.185	0.236	0.669
4	Lutein and Zeaxanthin	1.095	1.112	0.926	0.940	1.268	0.069	1.037	1.195	1.133	0.730
5	Lycopene	1.527	1.728	0.519	1.610	1.645	0.907	1.733	1.476	1.592	0.765
	Total	6.529	8.628	**0.008**	7.076	8.081	0.198	7.55	7.753	7.347	0.939

^1^*p*-Value < 0.05 means that the daily intake of a particular carotenoid differs significantly between the respective population groups. Bold numbers: *p*-Value < 0.05.

## Data Availability

Data will be made available upon request, by author prof. Nuri Andarwulan.

## Supplementary Materials

The following are available online at https://www.mdpi.com/article/10.3390/antiox10040587/s1, Table S1: questionnaire, respondents’ characteristics; Table S2: food recall 2x24 h questionnaire; Table S3: food frequency questionnaire; Table S4: list of foods used in food frequency interview; Table S5: list of all food products consumed by the respondents; Table S6: comparison of each food category’s consumption by different respondents’ groups (by location, gender, and age), Table S7: total flavonoid content of each food category, Table S8: total carotenoid content of each food category, Table S9: example of the flavonoid content calculation of the multi-ingredient food based on recipe elaboration, Table S10: example of carotenoid content calculation of the multi-ingredient food based on recipe elaboration, Table S11: list of food items with no relevant data on flavonoid content and their share (%) in total consumption, Table S12: list of food items with no relevant data on carotenoid content and their share (%) in total consumption, Table S13: comparison of the flavonoid intakes of different respondents’ groups, Table S14: comparison of the carotenoid intakes of different respondents’ groups.
