# Supplementary material for: Antioxidants Such as Flavonoids and Carotenoids in the Diet of Bogor, Indonesia Residents
Source: Antioxidants (Basel). 2021 Apr 11;10(4):587. doi: 10.3390/antiox10040587 (PMC8069321; doi:10.3390/antiox10040587)
Supplement: Supplementary file 1 [file antioxidants-10-00587-s001.pdf]

Supplementary Materials for the Article

Antioxidants such as Flavonoids and Carotenoids in the Diet of Bogor-Indonesia Residents

Nuri Andarwulan [1,2], Niken Cahyarani Puspita [1], Saraswati [1] and Dominika Średnicka-Tober [3,*]

[1] Department of Food Science and Technology, Faculty of Agricultural Technology, IPB University, P.O. Box 220, IPB Darmaga Campus, Bogor 16680, West Java, Indonesia; andarwulan@apps.ipb.ac.id (N.A.), sesilianiken.cp@gmail.com (N.C.P.), ginasaraswati@gmail.com (S.)

[2] Southeast Asian Food and Agricultural Science and Technology (SEAFAST) Center, IPB University, IPB Darmaga Campus, Bogor 16680, West Java, Indonesia

[3] Department of Functional and Organic Food, Institute of Human Nutrition Sciences, Warsaw University of Life Sciences, Nowoursynowska 159c, 02-776 Warsaw, Poland

* Correspondence: dominika_srednicka_tober@sggw.edu.pl; Tel.: +48225937035 (D.Ś.-T.).

List of Tables

Table S1. Questionnaire: respondents' characteristics. 2
Table S2. Food recall 2x24 hour questionnaire. 4
Table S3. Food frequency questionnaire. 7
Table S4. List of foods used in food frequency interview. 10
Table S5. List of all food products consumed by the respondents. 12
Table S6. Comparison of each food category consumption by different respondents' groups (representing different areas of residence, genders and age groups). 14
Table S7. Total flavonoid content of each food category. 17
Table S8. Total carotenoid content of each food category. 18
Table S9. Example of flavonoid content calculation of the multi-ingredient food based on recipe elaboration. 19
Table S10. Example of carotenoid content calculation of the multi-ingredient food based on recipe elaboration. 20
Table S11. List of food items with no relevant data on flavonoid content and their share (%) in total consumption. 21
Table S12. List of food items with no relevant data on carotenoid content and their share (%) in total consumption. 22
Table S13. Comparison of flavonoids intakes of different respondents' groups. 23
Table S14. Comparison of carotenoids intakes of different respondents' groups. 25

Table S1. Questionnaire: respondents' characteristics.

General Information about Respondent

1. Respondent Number : ________________________
2. Full name : ________________________
3. Gender : ________________________
4. Home Address : Street. _______________ No. ______
RT______ RW______
Sub-district_____________ District______________
5. Area : 1. City 2. Regency
6. Phone Number : ________________________
7. Interview date : ________________________2012
8. Enumerator : ________________________
9. Signature of enumerator : ________________________
10. Willing to draw blood (from the fingertip): 1. Yes 2. No

A. Respondent and Family Characteristics

1	Respondent No.	:
2	Full name	:
3	Age	:
4	Education	:
5	Occupation	:
6	Name of spouse*)	:
7	Age of spouse*)	:
8	Education of spouse*)	:
9	Occupation of spouse*)	:
10	Number of family members	:
11	Wife income (IDR/month)	:
	Husband income (IDR/month)	:
	Other family member income (IDR/month)	:
	Total family income	:
12	Total expenses (IDR/month)	:

*) cross the unnecessary ones

B. Respondent's Health Condition

1. Anthropometry
Body weight : …………… kg
Body height : …………… cm
2. Blood pressure : ……………
3. Current medical history:

No	Type of disease	Yes	No	Type of treatment	Note
1	High blood pressure				
2	Cardiovascular disease (CVD)				
3	High blood cholesterol				
4	Diabetes type I/II				
5	Kidney disease				

6	Liver disease				
7	High uric acid				
8					
9					
10					

4. Past medical history

No	Type of disease	Yes	No	Type of treatment*	Note**
1	Heart attack				
2	Stroke				
3	Liver disease				
4	Kidney disease				
5					
6					
7					
8					

*the name of drugs given (including traditional drugs/therapy), since when?
**ever been treated? Operation? specific types of disease

5. Family medical history

No	Type of disease	Yes	No	Family relation	Note**.
1	High blood pressure				
2	CVD				
3	High blood cholesterol				
4	Diabetes				
5	Kidney disease				
6	Obesity				
7					
8					

6. Beverages/foods consumed for health purposes …….
7. Beverages/foods restricted for health purposes …….

C. Habits of Smoking and Drinking Alcohol

No	Habits	Yes	No	Number/Frequency	Type	Since when?
1	Smoking				a. Filter/……………. b. Non Filter/………..	
2	Alcohol					

Table S2. Food recall 2x24 hour questionnaire**.**

General Information about Respondent

1. Respondent Number : ________________________
2. Full name : ________________________
3. Gender : ________________________
4. Home address : Street. ______________ No. ______ RT______ RW______
   Sub-district _____________ District______________
5. Area : 1. City 2. Regency
6. Phone Number : ________________________
7. Interview date : ________________________2012
8. Enumerator : ________________________
9. Enumerator signature : ________________________

Day 1 (Date: .d....../.m............./ .y…….)

Code[0]	Food Name[1]	Composition[2]	Food source		Portion	
			Buy[3] (place/brand)	Cook[4] (Processing technique)	Household size[5]	weight (g)[6]
	**Breakfast**					
	Rice cake with chayote soup	Rice cake<br>Coconut milk<br>Chayote<br>Egg	Buy		1 plate:<br>15 slices<br>2 tablesp<br>2 tablesp<br>1 pc	150<br>20<br>20<br>65
	**Snack**					
	Honeydew	Honeydew	Buy		1 slice	200
	**Lunch**					

	**Snack**					
	**Dinner**					
	**Midnight meal/snack**					
	**Supplement consumption**					

Day 2 (Date: .d....../.m............./ .y…….)

**Code[0]**	**Food Name[1]**	**Composition[2]**	**Food source**		**Portion**	
			**Buy[3] (place/brand)**	**Cook[4] (Processing technique)**		
	**Breakfast**					
	**Snack**					
	**Lunch**					
	**Snack**					
	**Dinner**					
	**Midnight meal/snack**					

	**Supplement consumption**					

Note

0 = Food code in enumerator database

1 = Food name

2 = Food composition, especially in multi-ingredient food

3 = Food source, buy. Write shop/restaurant where the product is bought. If the product has a brand, write the brand

4 = Food source, cook. Write the processing technique applied. If not cooked, write a dash

5 = Portion, in household size

6 = Portion, in gram

Table S3. Food frequency questionnaire.

General Information about Respondent

1. Respondent Number : ________________________
2. Full name : ________________________
3. Gender : ________________________
4. Home address : Street. _______________ No. ______ RT______ RW______
   Sub-district _____________ District______________
5. Area : 1. City 2. Regency
6. Phone Number : ________________________
7. Interview date : ________________________2012
8. Enumerator : ________________________
9. Enumerator signature : ________________________

Code[0]	Food type[1]	Composition[2]	Food source		Frequency[5] (times/week or times/month)	Portion	
			buy[3] (place/brand)	cook[4] (processing technique)		Household size[6]	Weight (g)[7]
**A**	**Cereals and cereal products**						
	Cooked white rice	White rice<br>Water		Cooked (by ricecooker)	90x/month	2 scoop of rice:<br>1 glass<br>2 glass	 100<br>200
**B**	**Tubers and tuber products**						
	Potato patties	Potato<br>Egg<br>Celery<br>Salt<br>Palm oil		Fried	3x/week	2 pcs medium:<br>2 pcs medium<br>1 tablesp<br>2 sheets<br>½ teasp<br>3 tablesp	 50<br>10<br>2<br>2.5<br>30

**C**	**Legumes and legume products**						
	Roasted peanuts	Peanuts<br>Salt		Roasted	4x/month	2 handfuls<br>1 teasp	100<br>5
**D**	**Vegetables and vegetable products**						
	Spinach soup	Spinach<br>Sweet corn<br>Shallot<br>Salt<br>Water		Boiled	10x/month	1 bowl :<br>1 handfuls<br>1 tablesp<br>2 cloves<br>1 teasp<br>1 glass	 15<br>10<br>5<br>5<br>200
E	**Fruits and fruit products**						
	Apple		Buy	-	3x/month	1 pc medium	150
**F**	**Herbs, spices, and condiments**						
	Sambal (chili sauce)	Cayenne pepper<br>Shallot<br>Garlic<br>Salt<br>Tomato		fried	5x/week	1 tablesp :<br>2 cloves<br>1 clove<br>½ teasp<br>1 pc small	 7<br>3<br>2.5<br>5
**G**	**Sweets and beverages**						
	Chocolate	Dark chocolate	Buy (silver queen dark chocolate)		2x/month	1 small slice	33
**H**	**Phytonutrient-fortified products**						
	Milk	Powdered milk<br>Water	Buy (Tropicana Slim non-fat phytosterol)	Dissolved in hot water	7x/week	2 tablesp<br>1 glass	20<br>200
**I**	**Supplements**						
**J**	**Fried food**						
	Fried coated tempeh	Tempeh<br>Wheat flour<br>Salt<br>Palm oil	Buy		3x/week	2 medium slices:<br>2 slices<br>1 tablesp<br>1 teasp<br>2 tablesp	 30<br>10<br>5<br>20
**K**	**Bakery/cake**						

	Biscuit		Buy (Khong Guan)		2x/week	3 pcs	30
**L**	**Food with peanut sauce**						
	Gado-gado	Spinach<br>Chayote<br>Carrot<br>Bean sprout<br>Peanut sauce<br>Soy sauce<br>Rice cake	Buy		3x/month	1 serving:<br>1 tablesp<br>1 tablesp<br>1 tablesp<br>1 tablesp<br>3 tablesp<br>1 tablesp<br>10 small slices	10<br>10<br>10<br>10<br>30<br>10<br>100
**M**	**Mixed food**						
	Soto ayam bening (Chicken soup with vegetables)	Chicken<br>Palm oil<br>Tomato<br>Celery<br>Salt<br>Water	Buy		4x/month	1 bowl:<br>1 small slice<br>0.5 tablesp<br>2 slices<br>2 sheets<br>¼ teasp<br>1 glass	15<br>5<br>5<br>2<br>2<br>150

Table S4. List of foods used in food frequency interview.

Code	Food Name	Code	Food Name
**A**	**Cereals and cereal products**	**B**	**Tubers and tuber products**
1	White rice	1	Potato
2	Brown rice	2	Cassava
3	Red rice	3	Taro
4	Sticky rice	4	Sweet potato
5	Black sticky rice	5	Potato chips
6	Corn	6	Cassava chips
7	Oatmeal	7	Sweet potato/taro chips
8	Instant noodle	8	Potato patties
9	Rice noodle		etc.
10	Pasta (spaghetti, macaroni, etc.)		
11	Breakfast cereal		
	etc.		
**C**	**Legumes and legume products**	**D**	**Vegetables and vegetable products**
1	Peanut	1	Katuk leaves/sweet leaves
2	Jack bean	2	Water spinach
3	Mung bean	3	Tomato
4	Soybean	4	Carrot
5	Red bean	5	Bamboo shoots
6	Peas	6	Chayote
7	Cashew nuts	7	Common bean
8	Tempeh	8	Cassava leaves
9	Tofu	9	Pak Choy
10	Oncom (fermented soybean/peanut sediment)	10	Lettuce
11	Soy milk	11	Cabbage
12	Mung bean drink	12	Cauliflower
13	Peanut butter	13	Broccoli
	etc.	14	Basil leaves
		15	Pepper
		16	Cucumber
		17	Bean sprout
		18	Eggplant
		19	Mushroom
			etc.
**E**	**Fruits and fruit products**	**F**	**Herbs, spices, and condiments**
1	Avocado	1	Sambal/chili sauce
2	Grape	2	Peanut sauce
3	Apple	3	Sweet soy sauce
4	Durian	4	Salty soy sauce
5	Orange	5	Tomato sauce
6	Persimmon	6	Vinegar
7	Mango	7	Ginger
8	Pineapple	8	Turmeric
9	Nutmeg	9	Peanut paste
10	Papaya	10	Margarine
11	Banana	11	Mayonnaise
12	Rambutan	12	Olive oil
13	Watermelon		etc.

Code	Food Name	Code	Food Name
14	Soursop		
15	Strawberry		
16	Honeydew		
17	Pear		
18	Guava		
	etc.		
**G**	**Sweets and beverages**	**H**	**Phytonutrient-fortified product**
1	Chocolate	1	TROPICANA SLIM non-fat fitosterol
2	Jelly	2	Nutrive BENECOL
3	Coffee		etc.
4	Tea		
	etc.		
**I**	**Supplements**	**J**	**Fried food**
1	Chlorophyll	1	Fried coated tempeh
2	Vitamin C	2	Fried coated tofu
	etc.	3	Fried tofu stuffed with vegetables
		4	Vegetable fritters
		5	Rissoles
		6	Crispy fried chicken
		7	Fried egg
		8	Banana fritters
			etc.
**K**	**Bakery/cake**	**L**	**Food with peanut sauce**
1	Biscuit	1	Gado-gado
2	Bakery	2	Siomay/steamed dumpling
3	Sandwich	3	Rujak (mixed fruits) with chili sauce
4	Cake	4	Doclang (rice cake, tofu, peanut sauce)
5	Cookies	**M**	**Mixed food**
6	Pan-fried bread (martabak)	1	Vegetables in tamarind soup
7	Bowsprit (kue cucur)	2	Mixed vegetables soup
8	Kue apem (steamed rice flour dough)	3	Capcay
9	Kue pancong (rice flour and coconut milk cake, with grated coconut)	4	Salad
10	Getuk (steamed mashed cassava)	5	Soto (chicken/meat soup with vegetables)
	etc.	6	Rice cake with vegetables
		7	Meatball soup
		8	Iced fruit cocktail

Table S5. List of all food products consumed by the respondents.

No	Food Group	Food products
1	Beverages	Chocolate milk powder; Coffee (black); Coffee (with milk); ENERGEN instant cereal drink; Es Doger (shaved ice with sweetened condensed milk, fermented black sticky, and jackfruit); Full cream milk powder; Liquid milk; Soft drinks; Sweetened condensed milk; Syrup; Tea without sugar; Tea with sugar
2	Fruits and fruit products	Apple, raw; Avocado, raw; Avocado, raw, with sugar; Banana, raw; Candied fruits; Durian; Grapes, raw; Honeydew, raw; Iced fruit cocktail dessert; Jackfruit, raw; Jicama, raw; Lime, raw; Longan, raw; Mango, raw; Orange, raw; Papaya, raw; Pear, raw; Persimmon, raw; Petis (Fruit salad with brown sugar sauce and tamarind); Pineapple, raw; Rambutan, raw; Rujak (Fruit salad with peanut sauce); Salak/snake fruit, raw; Sawo/sapodilla, raw; Sliced tomato, with sugar; Soursop, raw; Soursop, raw, with sugar; Star fruit, raw; Strawberry Jam; Strawberry, raw; Strawberry, raw, with sugar; Water apple, raw; Watermelon, raw; White guava, raw; White guava, raw, with sugar; Young coconut meat
3	Herbs, spices, and condiments	Bandrek (Ginger drink with coconut milk and brown sugar); Chili and peanut sauce with seasonings; Chili sauce; Chili sauce with seasonings, fried (sambal); Ginger extract drink; Jamu (mixed extract of medicinal plants); Soy sauce; Soy sauce + chopped cayenne pepper, with seasonings; Soybean paste (tauco); Tamarind and turmeric extract drink; Tomato sauce; Turmeric, raw; Vinegar
4	Cereals and cereal products	Batagor (fried dimsum with peanut sauce); Boiled corn; Boiled corn with grated coconut; Boiled vermicelli rice; Brown rice with chocolate flavor, boiled; Chicken porridge; Chocolate-flavored whole grain breakfast cereal; Fried noodle with soy sauce and vegetables; Fried rice; Fried sticky rice; Grilled corn on the cob; Instant fried noodle; Instant noodle soup; Ketoprak (rice cake, tofu, sprouts, peanut sauce); Kwetiaw goreng dengan sayuran dan kecap; Noodle with vegetable soup; Oatmeal; Pasta; Rice cake with coconut milk and vegetables; Rice cake with oncom/fermented soybean sediment; Rice cake with peanut sauce; Rice cooked in coconut milk/fragrant rice with side dish (nasi uduk); Spring rolls; Steamed black sticky rice; Steamed sticky rice with grated coconut; Stir-fried vermicelli rice; White rice
5	Eggs and egg products	Omelette; Sunny side up eggs
6	Fish and fish products	Fried common carp with spice; Fried salted fish<br>Fried salted tuna; Fried tilapia with spice
7	Legumes, legume products and nuts	Boiled peanuts; Boiled red beans; Boiled soybeans; Coated peanuts; Fried peanuts; Fried tempeh with soy sauce; Fried tempeh with soy sauce; Fried tofu; Fried tofu in soy sauce; Fried tofu with seasonings and tamarind sauce; Jack bean; Javanese sweet marinated tempeh; Kuaci (sunflower seed); Mung bean drink; Mung bean sweet porridge; Peanut butter; Peanuts with fried salted fish; Peas; Pepes tahu (spiced steamed tofu, wrapped in banana leaves); Red bean soup; Roasted cashews; Roasted soybeans; Sauteed common beans; Sauteed oncom; Sauteed tempeh with soy sauce; Soybean milk; Steamed oncom with spices; Stir-fried tofu; Tempeh with hot and spicy sauce (balado); Tofu in soy sauce; Tofu with hot and spicy sauce (balado)
8	Meat and meat products	Beef satay; Beef soup (meat/beef offal with spices); Beef soup with coconut milk (meat/beef offal with spices); Chicken braised in coconut milk; Chicken satay; Chicken soup; Chicken soup with coconut milk; Crispy fried chicken; Lamb satay; Meatballs soup with noodle and vegetables; Seasoned chicken noodle soup; Seasoned fried chicken
9	Snacks	Bakpia (bean-filled moon cake); Banana fritter; Biscuits with cream cheese; Biscuits with fruit jam; Biscuits; Bowsprit cake; Cake; Cheese sandwich; Chocolate Banana Crepes; Chocolate stuffed bread; Chocolate wafers; Cireng (fried tapioca dough); Combro (cassava dough with oncom and spices, fried); Cookies; Crackers; Cream cheese; Deep fried vegetables fritter; Deep fried-batter loaded with anchovies (rempeyek); Deep fried-batter loaded with peanuts (rempeyek); French fries; Fried banana chips; Fried battered-oncom; Fried battered-tempeh; Fried bread filled with fruit jam or chocolate; Fried cassava; Fried cassava chips; Fried cassava dough; Fried corn fritter; Fried donuts with margarine and chocolate meises; Fried sweet potato chips; Fried sweet potato dough; Fried sweet potatoes; Fried taro; Fried tofu; Fried

		vegetable croquettes; Fried-battered tofu stuffed with vegetables; Fruit jam filled bread; Getuk (steamed mashed cassava with brown sugar and grated coconut); Green bean sandwich; Ketimus (steamed cassava); Kue ape (traditional pancake from Jakarta); Kue apem (steamed rice flour dough); Kue lopis (sweet cake made of glutinous rice, steamed); Kue pancong (rice flour and coconut milk cake, with grated coconut); Lapis Legit (thousand layer cake); Margarine sandwich and meises; Meises; Milk chocolate; Minced meat bun; Misro (cassava dough with brown sugar, fried); Nagasari (steamed rice flour cake with banana); Pancakes, with beans, meises and sugar; Pancakes, with cheese and sweetened condensed milk; Pancakes, with sticky rice and sweetened condensed milk; Peanut butter cookies; Potato chips; Pudding; Pukis (coconut milk cake); Rengginang (sticky rice cracker); Rissoles filled with eggs and vegetables; Snack of fried rice flour; Taro chips, fried; Traditional snack made from fried wheat flour; Wheat flour snack with eggs and vegetables
10	Supplements	Chlorophyll; Vitamin C
11	Vegetables and vegetable products	Asinan (vegetables brined in vinegar and salt solution); Bamboo shoots, raw; Bamboo shoots, stir-fried; Basil, raw; Bitter apple, raw; Bitter gourd, sauteed; Broccoli, raw; Cabbage, raw; Candil (boiled sweet potato dough with brown sugar and coconut milk); Capcay (stir-fried vegetable mix); Carrots, raw; Carrots, sauteed; Cassava leaves soup; Cassava leaves, boiled; Cassava, steamed; Chayote, sauteed; Chayote, steamed; Chinese cabbage soup; Chinese cabbage, raw; Chinese cabbage, stir-fried; Chinese okra soup; Common beans, boiled; Common beans, stir-fried; Cucumber, raw; Cucumber, sauteed; Eggplant, sauteed; Fern, sauteed; Gado-gado (mixed boiled vegetables served with peanut sauce and tempeh); Getuk (steamed mashed cassava); Green lettuce, raw; Hot and spicy eggplant; Jackfruit with coconut milk and seasoning, boiled; Karedok (mixed raw vegetables served with peanut vegetable); Laksa (spicy rice noodle soup with bean sprouts); Lodeh (mixed vegetables soup with coconut milk); Long beans, stir-fried; Mashed taro with grated coconut; Melastome clearweed; Mixed boiled vegetables with grated coconut; Mixed vegetables in tamarind soup; Mixed vegetables soup, clear broth; Mushroom fries; Mushroom soup; Papaya leaves, boiled; Pecel (mixed green vegetables served with peanut sauce); Peppers, sauteed; Pickles; Potato patties; Potatoes, steamed; Salad; Spicy fried potato; Spinach fries; Spinach soup with corn; Spinach, boiled; Sweet leaf soup; Sweet potato patties; Sweet potato, boiled; Tapai (fermented cassava); Taro, steamed; Tauge goreng (bean sprout, tofu, and fermented soybean paste); Tomatoes, raw; Vegetable soup with chicken/meat/meatballs; Vegetables, stir-fried; Water spinach, sauteed; Yellow bur head, sauteed

Table S6. Comparison of each food category consumption by different respondents' groups (representing different areas of residence, genders and age groups).

**Category**	**Respondents Number (N=200)**	**Food Consumption (gram/person/day)**											
		**Beverages**	**Fruits and fruit products**	**Herbs, spices, and condiments**	**Cereals and cereal products**	**Eggs and egg products**	**Fish and fish products**	**Legumes and legume products**	**Meat and meat products**	**Snacks**	**Supplements**	**Vegetables and vegetable products**	**Total**
All	200	16.204	106.130	13.009	397.942	9.722	1.860	81.552	28.404	96.152	0.084	140.933	891.991
City	100	18.597	111.910	13.941	383.209	10.862	2.348	84.540	32.679	98.382	0.118	132.457	889.043
District	100	13.811	100.350	12.077	412.675	8.581	1.372	78.563	24.128	93.922	0.050	149.409	894.939
p-value		0.239	0.459	0.413	0.183	0.404	0.159	0.549	0.068	0.715	0.323	0.265	0.912
All, based on gender													
Female	100	18.212	108.672	17.134	371.564	9.873	1.980	85.710	27.920	93.105	0.164	152.761	887.094
Male	100	14.196	103.588	8.883	424.320	9.570	1.740	77.394	28.887	99.199	0.004	129.105	896.887
p-value		0.299	0.745	**0.004**	**0.011**	0.912	0.723	0.374	0.841	0.608	**0.019**	0.083	0.849
All, based on age													
25-40	102	14.003	104.337	14.994	421.286	8.267	1.648	77.852	31.168	100.690	0.095	136.639	910.978
41-55	63	19.704	109.333	11.055	394.886	13.906	1.426	87.014	28.119	90.516	0.113	151.411	907.484
56-65	35	16.317	105.590	10.740	335.412	6.429	3.259	82.501	20.862	93.071	0.000	134.588	808.767
p-value		0.455	0.958	0.389	**0.017**	0.067	0.144	0.683	0.294	0.722	0.507	0.605	0.299
All, Female, based on age													
25-40	57	18.126	111.278	19.982	395.679	7.007	1.794	81.775	32.255	104.607	0.165	163.912	936.579
41-55	29	16.910	110.594	13.678	350.393	17.959	2.318	101.919	26.156	85.580	0.241	156.064	881.812
56-65	14	21.257	94.083	12.698	317.235	4.795	2.036	68.152	13.927	61.861	0.000	100.516	696.562
p-value		0.982	0.851	0.467	0.094	**0.012**	0.908	0.248	0.177	0.071	0.544	0.093	**0.038**
All, Male, based on age													
25-40	45	8.781	95.545	8.676	453.721	9.863	1.464	72.884	29.791	95.729	0.006	102.092	878.551
41-55	34	22.088	108.258	8.818	432.837	10.449	0.664	74.301	29.794	94.726	0.004	147.442	929.381
56-65	21	13.024	113.261	9.434	347.529	7.517	4.074	92.066	25.484	113.877	0.000	157.303	883.571

Category	Respondents Number (N=200)	Food Consumption (gram/person/day)											
		Beve-rages	Fruits and fruit products	Herbs, spices, and condiments	Cereals and cereal products	Eggs and egg products	Fish and fish products	Legumes and legume products	Meat and meat products	Snacks	Supple-ments	Vegetables and vegetable products	Total
p-value		**0.048**	0.799	0.969	0.051	0.833	**0.008**	0.497	0.877	0.736	0.756	0.051	0.829
City, based on gender													
Female	50	27.394	106.067	18.635	354.782	8.777	2.401	85.553	29.094	100.600	0.228	145.191	878.722
Male	50	9.800	117.753	9.246	411.636	12.947	2.294	83.528	36.264	96.165	0.008	119.723	899.364
p-value		**0.007**	0.524	**0.023**	**0.023**	0.148	0.921	0.895	0.237	0.806	**0.017**	0.144	0.736
District, based on gender													
Female	50	9.029	111.277	15.633	388.346	10.969	1.558	85.866	26.747	85.610	0.100	160.330	895.466
Male	50	18.593	89.423	8.520	437.005	6.193	1.186	71.260	21.510	102.233	0.000	138.488	894.411
p-value		**0.027**	0.279	0.110	0.176	0.119	0.595	0.237	0.458	0.293	0.322	0.317	0.989
City, All, based on age													
25-40	46	17.889	95.317	14.264	388.917	11.119	2.137	74.543	36.613	103.959	0.210	127.274	872.243
41-55	33	21.207	136.711	16.215	412.751	12.231	1.243	104.696	34.980	106.119	0.065	153.793	1000.011
56-65	21	16.046	109.285	9.658	324.282	8.148	4.544	74.766	20.446	74.007	0.000	110.280	751.462
p-value		0.833	0.256	0.501	0.059	0.758	0.093	0.152	0.126	0.344	0.153	0.227	**0.021**
City, Female, based on age													
25-40	27	25.881	90.956	19.560	386.149	7.704	2.799	73.484	34.925	108.384	0.348	150.131	900.322
41-55	15	25.069	125.728	20.543	339.506	12.969	2.256	115.909	27.679	104.474	0.133	162.423	936.690
56-65	8	36.858	120.204	11.933	277.559	4.542	1.333	69.368	12.064	67.061	0.000	96.210	697.132
p-value		0.770	0.515	0.715	0.110	0.494	0.835	0.149	0.091	0.374	0.307	0.197	0.174
City, Male, based on age													
25-40	19	6.532	101.514	6.739	392.850	15.972	1.198	76.048	39.012	97.671	0.014	94.795	832.343
41-55	18	17.989	145.863	12.607	473.788	11.617	0.400	95.353	41.063	107.490	0.007	146.601	1052.778
56-65	13	3.238	102.565	8.258	353.035	10.367	6.520	78.087	25.604	78.282	0.000	118.939	784.896

Category	Respondents Number (N=200)	Food Consumption (gram/person/day)											
		Beve-rages	Fruits and fruit products	Herbs, spices, and condiments	Cereals and cereal products	Eggs and egg products	Fish and fish products	Legumes and legume products	Meat and meat products	Snacks	Supple-ments	Vegetables and vegetable products	Total
p-value		0.057	0.471	0.288	**0.030**	0.743	**0.001**	0.706	0.453	0.718	0.655	0.305	0.064
District, All, based on age													
25-40	56	10.811	111.746	15.593	447.875	5.924	1.246	80.571	26.694	98.005	0.000	144.331	942.796
41-55	30	18.052	79.218	12.362	375.236	15.748	1.626	67.564	20.573	73.352	0.167	148.790	805.705
56-65	14	16.724	100.048	12.362	352.105	3.850	1.332	94.103	21.485	121.666	0.000	171.049	894.724
p-value		0.357	0.386	0.113	0.059	**0.009**	0.900	0.342	0.719	0.140	0.314	0.703	0.261
District, Female, based on age													
25-40	30	11.146	129.567	20.361	404.256	6.380	0.889	89.237	29.851	101.208	0.000	176.315	969.210
41-55	14	8.168	94.379	6.321	362.057	23.305	2.385	86.930	24.524	65.337	0.357	149.251	823.015
56-65	6	0.456	59.256	13.717	370.137	5.133	2.974	66.532	16.412	54.928	0.000	106.257	695.801
p-value		0.393	0.270	0.303	0.615	**0.009**	0.387	0.732	0.737	0.054	0.282	0.330	0.113
District, Male, based on age													
25-40	26	10.424	91.184	10.091	498.204	5.399	1.658	70.572	23.052	94.310	0.000	107.425	912.318
41-55	16	26.700	65.953	4.555	386.767	9.135	0.961	50.619	17.115	80.366	0.000	148.387	790.558
56-65	8	28.925	130.642	11.346	338.582	2.888	0.100	114.782	25.290	171.720	0.000	219.644	1043.917
p-value		0.197	0.334	0.255	0.057	0.442	0.407	**0.012**	0.782	0.070	1.000	**0.019**	0.342

* p-value < 0.05 means that consumption level of particular food category differs significantly between respective population groups.

Table S7. Total flavonoid content of each food category.

No	Food group	Flavonoid content (mg/100 g)						
		Antho-cyanidin	Flavan-3-ol	Flavanone	Flavone	Flavonol	Isoflavone	Total
1	Beverages*	0.000	33.085	0.000	0.000	1.953	0.040	35.077
2	Fruits and fruit products	5.368	3.512	29.790	0.218	0.731	0.007	39.626
3	Herbs, spices, and condiments	1.979	3.120	0.000	9.549	3.794	12.753	31.194
4	Cereal and cereal products	0.284	0.078	0.601	1.278	1.883	2.181	6.305
5	Eggs and egg products	0.000	0.000	0.000	0.000	0.000	0.050	0.050
6	Fish and fish products	0.000	0.000	0.000	0.000	0.000	0.000	0.000
7	Legumes and legume products	1.209	7.760	0.984	8.121	6.409	24.554	49.036
8	Meat and meat products	0.294	0.168	1.336	0.032	2.763	0.003	4.596
9	Snacks	3.922	4.220	0.000	0.122	0.215	5.506	13.986
10	Supplements	0.000	0.000	0.000	0.000	0.000	0.000	0.000
11	Vegetables and vegetable products	0.945	2.010	0.525	1.369	10.022	0.088	14.959

*excluding drinking water.

Table S8. Total carotenoid content of each food category.

No	Food group	Carotenoid content (µg/100 g)					
		α-carotene	β-carotene	β-cryptoxanthin	Lutein & Zeaxanthin	Lycopene	Total
1	Beverages*	66.791	50.091	2.151	115.271	0.000	234.303
2	Fruits and fruit products	17.507	290.994	146.945	107.729	1845.653	2408.826
3	Herbs, spices, and condiments	62.935	258.565	12.155	194.199	7448.463	7976.317
4	Cereal and cereal products	60.912	175.935	35.634	220.149	1172.204	1664.835
5	Eggs and egg products	99.999	39.121	16.284	325.940	493.037	974.381
6	Fish and fish products	0.000	0.000	0.000	0.000	0.000	0.000
7	Legumes and legume products	59.381	158.499	6.642	300.389	572.804	1097.714
8	Meat and meat products	7.843	40.991	1.499	25.749	446.326	522.408
9	Snacks	215.458	972.504	14.029	32.757	9.000	1243.748
10	Supplements	0.000	0.000	0.000	0.000	0.000	0.000
11	Vegetables and vegetable products	363.077	1954.213	91.506	1521.539	282.540	4212.875

*excluding drinking water.

Table S9. Example of flavonoid content calculation of the multi-ingredient food based on recipe elaboration.

Food item	Ingredients	Flavonoid content in food ingredient (mg/100 g)							Proportion (%)	Flavonoid content in food item/dish (mg/100 g)[1]						
		**Anthocyanidin**	**Flavan-3-ol**	**Flavanone**	**Flavone**	**Flavonol**	**Isoflavone**	**Total Flavonoids**		**Anthocyanidin**	**Flavan-3-ol**	**Flavanone**	**Flavone**	**Flavonol**	**Isoflavone**	**Total Flavonoids**
Chicken soup	Seasoned chicken broth	0.072	0.000	0.000	0.005	0.386	0.000	0.463	31.70	0.023	0.000	0.000	0.002	0.122	0.000	0.147
	Chicken meat	0.000	0.000	0.000	0.000	0.000	0.000	0.000	30.65	0.000	0.000	0.000	0.000	0.000	0.000	0.000
	Fried onion	0.000	0.000	0.000	0.000	24.700	0.000	24.700	1.75	0.000	0.000	0.000	0.000	0.432	0.000	0.432
	Potato	0.000	0.000	0.000	0.000	1.500	0.000	1.500	26.27	0.000	0.000	0.000	0.000	0.394	0.000	0.394
	Tomato	0.000	0.000	0.000	0.010	0.720	0.000	0.730	8.76	0.000	0.000	0.000	0.001	0.063	0.000	0.064
	Celery	0.000	0.000	0.000	3.900	0.610	0.000	4.510	0.88	0.000	0.000	0.000	0.034	0.005	0.000	0.040
	**Total**									**0.023**	**0.000**	**0.000**	**0.037**	**1.017**	**0.000**	**1.077**[2]

[1]Flavonoid content of each ingredient was calculated based on this formula: flavonoid content of each ingredient x proportion of certain ingredient in food item

[2]Total flavonoid content of chicken soup was calculated based on this formula: total flavonoid content of seasoned chicken broth + total flavonoid content of chicken meat + total flavonoid content of fried onion + total flavonoid content of potato + total flavonoid content of tomato + total flavonoid content of celery

Total flavonoid intake from chicken soup (XI_i) = weight of chicken soup consumed x $\frac{\text{total flavonoid content of chicken soup}}{100}$ x $\frac{\text{edible portion}}{100}$

= 100 g x 1.077 mg/100 g x 100/100 = 107.7 mg

The estimated daily flavonoid intake from chicken soup = $\frac{XI_i \times f_i}{30 \text{ days}} = \frac{107.7 \text{ mg} \times 3}{30 \text{ days}} = 10.77$ mg/day

(Note: f_i is a frequency of chicken soup consumption in a month)

Table S10. Example of carotenoid content calculation of the multi-ingredient food based on recipe elaboration.

Food item	Food ingredients	Carotenoid content in food ingredient (μg/100 g)						Proportion (%)	Carotenoid content in food item/dish (μg/100g)					
		**α-carotene**	**β-carotene**	**β-cryptoxanthin**	**Lutein & zeaxanthin**	**Lycopene**	**Total carotenoids**		**α-carotene**	**β-carotene**	**β-cryptoxanthin**	**Lutein & zeaxanthin**	**Lycopene**	**Total carotenoids**
Pickles	Cucumber	11.0	45.0	26.0	23.0	0.0	105.0	31.25	3.4	14.1	8.1	7.2	0.0	32.8
	Carrot	3776.0	8332.0	0.0	194.0	n.a	12302.0	31.25	1180.0	2603.8	0.0	60.6	0.0	3844.4
	Chili	534.0	36.0	40.0	709.0	0.0	1319.0	12.50	66.8	4.5	5.0	88.6	0.0	164.9
	Onion	0.0	1.0	0.0	3.0	n.a	4.0	25.00	0.0	0.2	0.0	0.8	0.0	1.0
**Total**									**1250.2**	**2622.6**	**13.1**	**157.2**	**0.0**	**4043.1**

[1]Carotenoid content of each ingredient was calculated based on this formula: carotenoid content of each ingredient x proportion of certain ingredient in food item
[2]Total carotenoid content of pickles was calculated based on this formula: total carotenoid content of cucumber + total carotenoid content of carrot + total carotenoid content of chili + total carotenoid content of onion

Total carotenoid of pickles (XI_j) = weight of pickles consumed x $\frac{\text{total carotenoid content of pickles}}{100}$ x $\frac{\text{edible portion}}{100}$

= 10 g x 4043.1 μg/100 g x 100/100 = 40431 μg

The estimated daily carotenoid intake from pickles = $\frac{XI_j \times f_j}{30 \text{ days}}$ = $\frac{40431 \text{ μg} \times 5}{30 \text{ days}}$ = 6738.5 μg/day

(Note: f_j is frequency of pickles consumption in a month)

Table S11. List of food items with no relevant data on flavonoid content and their share (%) in total consumption.

No	Food Group	Name of Food Item	Food Consumption (g/person/day)	Food Consumption Percentage (%)
1	Beverages	Coffee, with milk	1.865	0.21
2	Cereals and cereal products	Oatmeal	0.732	0.08
3	Fruits and fruit products	Longan, raw	0.087	0.01
4	Fruits and fruit products	Rambutan, raw	0.168	0.02
5	Fruits and fruit products	Sapodilla, raw	0.124	0.01
6	Fruits and fruit products	Water apple, raw	0.139	0.02
7	Herbs, spices, and condiments	Chili sauce	0.606	0.07
8	Herbs, spices, and condiments	Margarine	0.225	0.03
9	Herbs, spices, and condiments	Ginger drink	0.100	0.01
10	Herbs, spices, and condiments	Bandrek (herbal drink with coconut milk)	0.083	0.01
11	Herbs, spices, and condiments	Herbal drink	4.561	0.51
12	Herbs, spices, and condiments	Tamarind with palm sugar drink	0.000	0.00
13	Legumes and legume products	Mung bean drink	1.208	0.14
14	Snacks	Biscuits with peanut butter	0.000	0.00
15	Snacks	Cheese sandwich	0.285	0.03
16	Snacks	Chocolate paste sandwich	3.363	0.38
17	Snacks	Fruit-based jam sandwich	0.203	0.02
18	Snacks	White bread with chocolate compound and margarine	1.505	0.17
19	Snacks	Mung bean sandwich	0.525	0.06
20	Snacks	Potato chips	0.550	0.06
21	Snacks	Sweet potato chips	0.050	0.01
22	Snacks	Chocolate wafer	0.198	0.02
23	Snacks	Getuk (Sweet steamed loaf of pounded cassava)	0.000	0.00
24	Supplements	Klorofil	0.025	0.00
25	Supplements	Enervon-C	0.000	0.00
26	Vegetables and vegetable products	Fermented cassava	0.072	0.01
27	Vegetables and vegetable products	Stir fried yellow bur head	0.046	0.01
	**Total**		**16.719**	**1.87**

Table S12. List of food items with no relevant data on carotenoid content and their share (%) in total consumption.

No	Food Group	Name of Food Item	Food Consumption (g/person/day)	Food Consumption Percentage (%)
1	Beverages	ENERGEN, Instant cereal drink	0.608	0.07
2	Cereals and cereal products	Steamed black sticky rice	0.105	0.01
3	Fruits and fruit products	Sapodilla, raw	0.124	0.01
4	Fruits and fruit products	Water apple, raw	0.139	0.02
5	Herbs, spices, and condiments	Tauco	0.054	0.01
6	Herbs, spices, and condiments	Bandrek (herbal drink with coconut milk)	0.083	0.01
7	Herbs, spices, and condiments	Herbal drink	0.000	0.00
8	Snacks	Biscuits with fruit jam	0.000	0.00
9	Snacks	Minced meat sandwich	0.000	0.00
10	Snacks	Fruit-based jam sandwich	0.203	0.02
11	Snacks	White bread with chocolate compound and margarine	1.505	0.17
12	Snacks	Mung bean sandwich	0.525	0.06
13	Snacks	Cassava chips	1.869	0.21
14	Supplements	Klorofil	0.025	0.00
15	Supplements	Enervon-C	0.000	0.00
16	Vegetables and vegetable products	Fermented cassava	0.000	0.00
17	Vegetables and vegetable products	Melastome clearweed, raw	0.011	0.00
	**Total**		**5.250**	**0.59**

Table S13. Comparison of flavonoids intakes of different respondents' groups.

Category	Respondents Number (N=200)	Flavonoid Intake (mg/person/day)						
		Total	Anthocyanidins	Flavan-3-ols	Flavanones	Flavones	Flavonols	Isoflavones
All	200	149.520	2.709	4.575	6.142	2.237	15.647	32.200
City	100	195.480	3.072	5.173	6.730	2.237	16.095	28.847
District	100	124.775	2.347	3.978	5.555	1.861	15.199	26.553
p-value		0.113	**0.026**	0.118	0.428	0.079	0.644	**0.047**
All, based on gender								
Female	100	155.481	2.756	4.758	5.793	3.418	16.929	30.519
Male	100	143.558	2.663	4.392	6.491	4.374	14.364	33.881
p-value		0.662	0.795	0.577	0.606	0.393	0.166	0.391
All, based on age								
25-40	102	161.246	2.549	3.824	6.750	4.785	14.477	30.914
41-55	63	140.995	2.761	5.434	6.110	2.801	18.045	33.135
56-65	35	130.692	3.083	5.217	4.428	3.275	14.740	34.262
p-value		0.695	0.584	0.100	0.500	0.310	0.198	0.765
All, Female, based on age								
25-40	57	162.270	2.702	3.909	6.758	4.309	16.705	31.164
41-55	29	157.094	3.104	5.336	5.220	2.432	18.319	31.097
56-65	14	124.501	2.254	7.016	3.055	1.833	14.964	26.694
p-value		0.703	0.650	0.105	0.297	0.239	0.671	0.836
All, Male, based on age								
25-40	45	159.948	2.355	3.716	6.741	5.389	11.655	30.598
41-55	34	127.263	2.469	5.519	6.869	3.116	17.810	34.874
56-65	21	134.819	3.636	4.018	5.344	4.237	14.590	39.307
p-value		0.836	0.122	0.259	0.879	0.629	0.138	0.462
City, based on gender								
Female	50	173.139	3.102	3.102	7.287	2.536	17.377	34.485
Male	50	175.390	3.043	3.043	6.173	1.938	14.812	36.620
p-value		0.962	0.859	0.859	0.475	0.069	0.234	0.743
District, based on gender								
Female	50	137.824	2.262	3.747	4.300	1.675	16.481	26.553
Male	50	111.727	2.175	4.208	6.810	2.047	13.916	31.141
p-value		0.363	0.890	0.557	0.258	0.212	0.400	0.303
City, All, based on age								
25-40	46	177.514	2.788	2.788	6.568	2.211	15.457	31.801
41-55	33	197.126	3.482	3.482	8.805	2.487	18.554	42.221
56-65	21	131.220	3.050	3.050	3.823	1.902	13.629	33.290
p-value		0.635	0.349	0.349	0.084	0.887	0.210	0.301
City, Female, based on age								
25-40	27	162.893	2.690	2.690	7.605	2.436	16.213	33.222
41-55	15	208.319	3.877	3.877	8.499	3.108	21.676	39.886

Category	Respondents Number (N=200)	Flavonoid Intake (mg/person/day)						
		Total	Anthocyanidins	Flavan-3-ols	Flavanones	Flavones	Flavonols	Isoflavones
56-65	8	141.755	3.035	3.035	3.939	1.801	13.250	28.621
p-value		0.396	0.260	0.260	0.403	0.152	0.121	0.703
City, Male, based on age								
25-40	19	198.291	2.927	2.927	5.094	1.891	14.383	29.782
41-55	18	187.798	3.153	3.153	9.060	1.970	15.952	44.167
56-65	13	124.737	3.060	3.060	3.751	1.964	13.862	36.164
p-value		0.815	0.942	0.942	0.167	0.986	0.848	0.314
District, All, based on age								
25-40	56	147.882	2.224	3.905	6.900	2.052	13.673	30.186
41-55	30	79.250	1.969	4.027	3.145	1.397	17.484	23.141
56-65	14	129.899	3.133	4.161	5.336	2.095	16.406	35.719
p-value		0.125		0.974	0.368	0.140	0.498	0.143
District, Female, based on age								
25-40	30	161.709	2.574	4.212	5.995	2.036	17.149	29.312
41-55	14	102.209	2.083	3.465	1.707	1.178	14.723	21.680
56-65	6	101.495	1.121	1.357	1.876	1.032	17.249	24.125
p-value		0.492	0.609	0.406	0.228	**0.016**	0.847	0.313
District, Male, based on age								
25-40	26	131.928	1.819	3.552	7.945	2.070	9.662	31.194
41-55	16	59.161	1.621	4.518	4.404	1.588	19.900	24.420
56-65	8	151.201	4.441	5.720	7.931	2.891	15.774	44.415
p-value		0.108	**0.044**	0.429	0.721	0.272	0.129	0.190

* p-value < 0.05 means that the flavonoids intake differs significantly between respective population groups.

Table S14. Comparison of carotenoids intakes of different respondents' groups.

Category	Respondents Number (N=200)	Carotenoid Intake (μg/person/day)					
		Total	α-carotene	β-carotene	β -cryptoxanthin	Lutein and Zeaxanthin	Lycopene
All	200.000	7578.404	563.826	4074.181	209.976	1103.610	1627.547
City	100.000	6529.306	517.528	3189.520	201.478	1094.988	1527.263
District	100.000	8627.502	610.124	4958.842	218.474	1112.232	1727.830
p-value		**0.008**	0.289	**0.000**	0.659	0.926	0.519
All, based on gender							
Female	100.000	8080.834	590.183	4334.354	243.806	1267.527	1644.957
Male	100.000	7075.974	537.470	3814.008	176.146	939.693	1610.136
p-value		0.198	0.515	0.276	0.081	0.069	0.907
All, based on age							
25-40	102.000	7549.602	584.009	3979.151	216.467	1036.840	1733.145
41-55	63.000	7753.413	592.018	4306.889	185.094	1195.468	1476.367
56-65	35.000	7347.325	454.264	3932.251	235.848	1132.852	1591.926
p-value		0.939	0.466	0.795	0.669	0.730	0.765
All, Female, based on age							
25-40	57.000	8391.050	659.755	4558.959	257.349	1073.789	1841.199
41-55	29.000	8408.959	583.378	4584.508	230.542	1680.211	1330.299
56-65	14.000	6138.120	321.021	2901.716	216.143	1201.476	1497.764
p-value		0.312	0.107	0.171	0.873	0.151	0.403
All, Male, based on age							
25-40		6483.767	488.063	3244.728	164.682	990.039	1596.277
41-55		7194.271	599.388	4070.096	146.330	782.010	1600.954
56-65		8153.461	543.092	4619.274	248.985	1087.103	1654.701
p-value		0.562	0.732	0.293	0.340	0.578	0.996
City, based on gender							
Female	50.000	6779.266	607.771	3472.415	235.410	1185.225	1278.432
Male	50.000	6279.346	427.285	2906.625	167.546	1004.751	1776.094
p-value		0.572	0.077	0.218	0.117	0.375	0.307
District, based on gender							
Female	50.000	9382.401	572.595	5196.293	252.202	1349.830	2011.482
Male	50.000	7872.602	647.654	4721.391	184.746	874.634	1444.178
p-value		0.245	0.551	0.575	0.300	0.114	0.097
City, All, based on age							
25-40	46.000	6192.036	539.103	2954.652	170.497	1025.612	1502.192
41-55	33.000	7717.233	634.123	4042.357	215.760	1156.139	1673.479
56-65	21.000	5401.347	287.049	2363.823	246.899	1150.861	1352.410
p-value		0.208	**0.047**	**0.031**	0.478	0.830	0.906
City, Female, based on age							
25-40	27.000	6433.750	655.943	3282.075	191.816	1060.226	1243.690

Category	Respondents Number (N=200)	Carotenoid Intake (µg/person/day)					
		Total	α-carotene	β-carotene	β -cryptoxanthin	Lutein and Zeaxanthin	Lycopene
41-55	15.000	8188.370	696.320	4498.325	266.229	1411.892	1315.563
56-65	8.000	5303.315	279.164	2191.232	324.759	1182.098	1326.062
p-value		0.272	0.263	0.059	0.490	0.679	0.985
City, Male, based on age							
25-40	19.000	5848.548	373.068	2489.365	140.203	976.425	1869.537
41-55	18.000	7324.619	582.292	3662.383	173.703	943.012	1971.743
56-65	13.000	5461.674	291.901	2470.033	198.985	1131.638	1368.625
p-value		0.608	0.054	0.298	0.693	0.810	0.881
District, All, based on age							
25-40	56.000	8664.746	620.895	4820.705	254.227	1046.063	1922.856
41-55	30.000	7793.212	545.703	4597.874	151.362	1238.729	1259.543
56-65	14.000	10266.292	705.087	6284.893	219.273	1105.840	1951.200
p-value		0.430	0.731	0.360	0.367	0.846	0.194
District, Female, based on age							
25-40	30.000	10152.621	663.186	5708.154	316.329	1085.995	2378.957
41-55	14.000	8645.305	462.370	4676.848	192.305	1967.696	1346.087
56-65	6.000	7251.194	376.831	3849.027	71.323	1227.314	1726.699
p-value		0.427	0.199	0.405	0.169	0.197	0.208
District, Male, based on age							
25-40	26.000	6947.967	572.098	3796.724	182.570	999.988	1396.586
41-55	16.000	7047.629	618.620	4528.772	115.536	600.884	1183.818
56-65	8.000	12527.615	951.278	8111.792	330.236	1014.734	2119.575
p-value		0.063	0.490	**0.028**	0.299	0.637	0.347

* p-value < 0.05 means that the carotenoids intake differs significantly between respective population groups.
